# Supplementary material for: Social Media Sentiment about COVID-19 Vaccination Predicts Vaccine Acceptance among Peruvian Social Media Users the Next Day
Source: Vaccines (Basel). 2023 Apr 7;11(4):817. doi: 10.3390/vaccines11040817 (PMC10146388; doi:10.3390/vaccines11040817)
Supplement: Supplementary file 1 [file vaccines-11-00817-s001.zip › vaccines-2267581-supplementary.pdf]

Online Supplementary Material for Social Media Amplification of Risk about COVID-19 Vaccination and Vaccine Acceptance Among Peruvian Social Media Users: A Longitudinal Time Series Analysis

S1. Topic Modeling

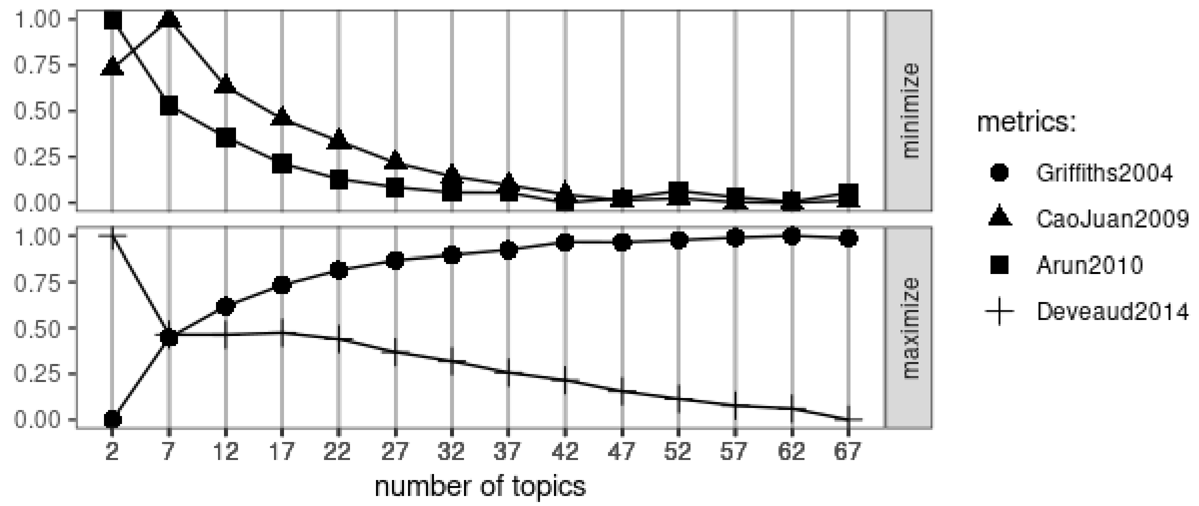

Figure S1 LDAtuning findk results plot

The visualization of finding the optimal number of topics using the four algorithms provided by the LDAtuning package [82].

Table S1 Top 10 Words for each topic and the assigned clusters

Cluster	*Topic Name*	*Top Words*
COVID Economy	Economy and the Pandemic	ninos, venezolanos, migrantes, personas, estan, colombia, miles, jovenes, padres, frontera
COVID Economy	COVID and Economy	pandemia, crisis, pais, politica, peor, mundo, situacion, medio, economica, economia
COVID Economy	Economy and the Pandemic	cambio, dolar, precio, mercado, precios, venezuela, peru, tipo, climatico, nuevo
COVID Health	COVID and Institutions	personas, ayuda, puedan, urgencia, instituciones, agradece, siguiente, favor, solicita, colaborar
COVID Health	Health Professionals and COVID	salud, COVID, hospital, personal, pacientes, oxigeno, medico, atencion, medicos, camas
COVID Non-Pharmaceutical Interventions	Non-Pharma COVID Precautions	mascarilla, puede, aire, hacer, usar, medidas, personas, mascarillas, seguir, pueden
COVID Non-Pharmaceutical Interventions	COVID	medidas, semana, clases, restricciones, COVID, pandemia, nuevas, gobierno, santa, cuarentena
COVID Non-Pharmaceutical Interventions	Lockdowns	millones, dolares, euros, dinero, pagar, soles, pago, pesos, empresas, casi
COVID Others	Latin America Pandemic	casos, COVID, nuevos, coronavirus, repo, horas, contagios, ultimas, fallecidos, registra
COVID Others	COVID	casos, COVID, datos, dias, mayor, aumento, espana, fallecidos, numero, cada
COVID Others	Global Pandemic English	people, need, know, just, health, dont, pandemic, like, says, many
COVID Others	Global Pandemic 2 English	year, china, the, global, climate, world, says, pandemic, years, billion
COVID Others	Governments during Pandemic	salud, impo, educacion, publica, ciencia, pandemia, ancia, mental, trabajo, desarrollo
COVID Others	COVID	COVID, estudio, virus, vacunas, riesgo, puede, enfermedad, ivermectina, personas, segun
COVID Vaccination	Vaccinations	vacunacion, mayores, anos, personas, COVID, adultos, dosis, poblacion, vacunar, proceso
COVID Vaccination	Vaccination	vacunas, dosis, millones, COVID, vacuna, pfizer, paises, pais, mexico, lote
COVID Vaccination	Vaccination	nueva, europa, unido, reino, paises, espana, york, coronavirus, union, europea
COVID Vaccination	Vaccinations	vacuna, dosis, COVID, astrazeneca, pfizer, primera, segunda, vacunas, coronavirus, sputnik
COVID Vaccination	Vaccination English	COVID, vaccine, cases, coronavirus, repo, people, deaths, vaccines, variant, million
COVID Variants	COVID Variants	COVID, variante, coronavirus, nueva, delta, positivo, virus, india, caso, china
International	US Protests	horror, laughing, like, house, heres, capitol, protrump, guns, stormed, doors

International	World News	mujeres, violencia, sexual, derechos, israel, humanos, genero, abuso, mujer, victimas
International	Global Politics	biden, trump, eeuu, presidente, unidos, casa, capitolio, donald, blanca, rusia
International	US Elections	biden, trump, president, says, house, white, former, election, first, trumps
International	US & UK News	police, people, death, killed, woman, prince, says, family, dead, found
International	World News	johnson, first, says, live, boris, minister, time, england, kong, tokyo
International	Global News (Miami Building Accident & Israel)	tras, menos, miami, personas, edificio, accidente, heridos, video, dejo, deja
International	News Headlines	luis, jose, albe, juan, fernandez, carlos, miguel, garcia, jorge, rodriguez
International	Olympics	juegos, tokio, olimpicos, final, depo, tras, mundial, gran, espana, historia
International	News Headlines	policia, video, seguridad, personas, banda, policias, detalles, armas, delincuentes, nacional
International	US Elections	agenda, open, georgia, much, senate, bidens, voting, determine, polls, controls
International	Politics Spain	madrid, real, sanchez, espana, gobierno, ayuso, directo, pablo, iglesias, tras
Other	Pop Culture News	madre, familia, hijo, padre, hija, vida, principe, reina, harry, tras
Other	Sports	fecha, liga, equipo, final, colo, boca, victoria, futbol, vivo, river
Other	Mixed News	detalles, aqui, saber, tener, puedes, hacer, info, conoce, debes, contamos
Other	Pop Culture	mundo, conve, tierra, espacio, primer, video, historia, primera, nuevo, irse
Other	Global Politics and News	anos, papa, historia, francisco, hace, gran, vida, celebra, amor, iglesia
Other	Sports	copa, america, final, messi, argentina, barcelona, seleccion, libe, brasil, futbol
Other	South America	ciudad, mexico, agua, metro, centro, depa, amento, capital, zona, linea
Other	Mixed Entertainment	vivo, programa, sigue, minuto, aqui, canal, senal, abie, directo, radio
Other	Peru Politics	elecciones, electoral, vuelta, segunda, resultados, votos, mesa, candidatos, voto, actas
Other	Entertainment	vivo, serie, nueva, cnmovista, nnen, cndirectv, cnbestcable, pelicula, netflix, mejor
Other	News Headlines	video, redes, sociales, fotos, foto, imagenes, viral, tras, hizo, compa
Other	Headlines Mix	noticias, hora, nacional, ultima, internacional, impo, peru, diario, compa, oficial

Peruvian Politics Election	Peruvian Elections	castillo, pedro, peru, keiko, fujimori, libre, cerron, popular, fuerza, equipo
Peruvian Politics Election	Peruvian Elections 2	lopez, candidato, presidente, aliaga, rafael, obrador, presidencial, soto, guzman, julio
Peruvian Politics Election	Social Media	america, cuenta, twitter, latina, whatsapp, facebook, informacion, google, traves, internet
Peruvian Politics Election	Peru Politics	keiko, izquierda, derecha, votar, voto, peru, solo, ahora, castillo, fujimori
Peruvian Politics General	Politics	ministro, presidente, gobierno, sagasti, salud, vivo, prensa, francisco, consejo, ministros
Peruvian Politics General	Politics - Constitution	congreso, comision, proyecto, presidente, constitucional, reforma, constitucion, mesa, pleno, popular
Peruvian Politics General	Politics	estan, medios, politicos, solo, comunicacion, ahora, hacen, haciendo, dicen, tambien
Peruvian Politics General	Peru Politics	vizcarra, dice, ahora, gobierno, solo, sagasti, senor, hizo, presidente, debe
Peruvian Politics General	Peru Politics	libe, democracia, pais, derecho, pueblo, debe, poder, peru, vamos, resion
Peruvian Politics General	Politics - Corruption	caso, fiscal, fiscalia, justicia, investigacion, prision, juez, judicial, corrupcion, tribunal
Regional Protests	South America Politics & Cuban Protests	cuba, colombia, protestas, presidente, duque, gobierno, venezuela, ivan, tras, haiti
Venezuela	Venezuela	gobierno, publico, sector, transpo, plan, nacional, empresas, trabajo, servicio, ministerio
Venezuela	Venezuelan Politics	maduro, venezuela, regimen, guaido, nicolas, presidente, detalles, alex, venezolanos, rodriguez

Among the COVID-related topics, COVID vaccination stood out most clearly as prominent, including 8.2% of the tweets, with the most temporal variability and the greatest standard deviation of prevalence (SD=.7%) followed by COVID Non-Pharmaceutical Interventions (e.g., lockdowns, masking, quarantines, travel restrictions) at 4.5%. The variances in topic prominence of other COVID-related topics such as Non-Pharmaceutical Interventions (SD=.3%) and COVID Health impacts (SD=.2%) was substantially less. The lower variation in non-vaccine COVID-related topics may harm the efforts to associate their prevalence with risk factors.

S2. Sentiment Analysis

The sentiment calculations were done using a crowdsourced sentiment lexicon in English and Spanish [89]. The lexicon is open-source and can be downloaded via the website https://saifmohammad.com/WebPages/NRC-Emotion-Lexicon.htm, or by the Tidyverse package [109] in R Studio. The lexicon works by giving a score to the words in the sentence, with corresponding sentiment and emotion. At Figure S2.1 you can see an example from the corpus and the sentiment scoring.

Figure S2 Sentiment analysis example

Tweet

- "bes falso que la vacuna china sinovac de coronavirus sea ineficaz de acuerdo con datos del ministerio de salud de chile la eficacia para evitar enfermedad supera el y el para evitar la mue"

NRC Dictionary in English and Spanish

- negative score: falso (5) + ineficaz (2) + evitar (2) + enfermedad (5) + evitar (2) = 16
- positive score: sea (1) + acuerdo (3)
- trust score: acuerdo (3) + ministerio (1) = 4

Net Sentiment

- Net sentiment: positive (5) - negative (16) = -11

There are multiple limitations to using a lexicon based approach to sentiment analysis, which Mohammad & Turney describe in detail in their paper introducing NRC [89]. First and foremost, lexicon approach does not take context into account, such as the sentence could be satirical, cynical, or a using word with local or temporal homonyms. Secondly, the word-sentiment scoring does not consider preceding and anterior words in sentiment calculations.

S3. Sentiment Analysis weighed with topic prominence

From the LDA object we receive for each document, in our case, tweet's gamma value which represents the probability of the topic in each of the documents. For each tweet we multiplied the gamma value of the tweet for the topic cluster Covid Vaccination with net sentiment and trust (figure S3.1).

Figure S3 Sentiment scores weighted with topic prominence example

Tweet

- "bes falso que la vacuna china sinovac de coronavirus sea ineficaz de acuerdo con datos del ministerio de salud de chile la eficacia para evitar enfermedad supera el y el para evitar la mue"

Sentiment Scores

- Net Sentiment: -11
- Trust: 4

Gamma Value for Covid Vaccination Cluster

- 0.6164384

Sentiment weighted with topic probability

- Net Sentiment Covid Vaccination: $-11 \times 0.6164384 = -6.7808224$
- Trust Sentiment Covid Vaccination: $4 \times 0.6164384 = 2.4657536$

S4. The Global COVID-19 Trends and Impact Survey Open Data API [4]

The dependent variable Vaccination Acceptance in Peru was retrieved from the API of The Global COVID-19 Trends and Impact Survey Open Data API [110]**.** The dependent variable was created with two separate API indicator names as the initial indicator was replaced in Wave 11:

- For 12/20/2020 to 05/19/2021 the indicator vaccine_acpt, defined as "Respondents definitely or probably choosing to get vaccinated if a COVID-19 vaccine was offered to them, out of the respondents who have not been vaccinated. Note: Replaced by appointment_or_accept_covid_vaccine in Wave 11"; and,
- For 05/20/2021 to 08/09/2021 the indicator vaccinated_appointment_or_accept, defined as "Respondents who had a vaccine, an appointment to get vaccinated or who would definitely or probably choosing to get vaccinated if a COVID-19 vaccine was offered to them" [18].

S4 Fig Sample Sizes of Vaccine Acceptance from The Global COVID-19 Trends and Impact Survey Open Data API [110]

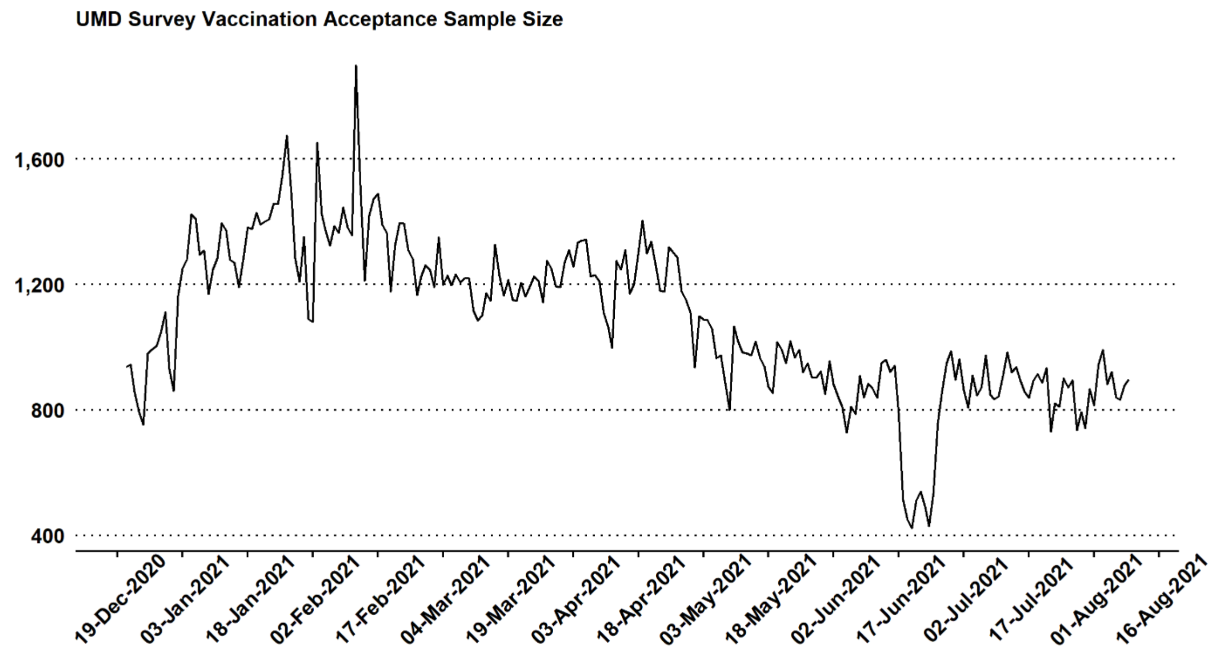

S5. Robustness Checks

Table S2 Reverse Time Series

	**Net Sentiment**		**Trust**	
	Estimates	*CI*	*Estimates*	*CI*
Vaccine Acceptance % (lag 1)	-0.036 (0.019)	[-0.073, 0.001]	-0.031 (0.018)	[-0.067, 0.005]
Vaccine Acceptance % (lag 2)	0.016 (0.019)	[-0.021, 0.053]	0.027 (0.018)	[-0.009, 0.064]
Vaccine Acceptance % (lag 3)	-0.033 (0.019)	[-0.070, 0.003]	-0.029 (0.018)	[-0.065, 0.007]
trend	0.000 (0.000)	[-0.000, 0.000]	-0.000 (0.000)	[-0.000, 0.000]
Observations	227		227	
R^2 / R^2 adjusted	0.416 / 0.392		0.556 / 0.538	

* *p<0.05* ** *p<0.01* *** *p<0.001*

Table S3 Time Series Analysis with Only Vaccinated Survey

	**covid_vaccine (v1)**	
Predictors	*Estimates*	*CI*
Trust (lag 1)	0.471 (-0.24)	[-0.002, 0.944]
Trust (lag 2)	-0.052 (-0.258)	[-0.561, 0.456]
Trust (lag 3)	0.039 (-0.239)	[-0.432, 0.509]
trend	0.000 * (0)	[0.000, 0.000]
Observations	212	
R^2 / R^2 adjusted	0.990 / 0.990	

* *p<0.05* ** *p<0.01* *** *p<0.001*

	**covid_vaccine (v1)**	
Predictors	*Estimates*	*CI*
Net Sentiment (lag 1)	0.274 (-0.247)	[-0.214, 0.761]
Net Sentiment (lag 2)	-0.079 (-0.248)	[-0.568, 0.411]
Net Sentiment (lag 3)	-0.251 (-0.242)	[-0.727, 0.226]
trend	0 (0)	[-0.000, 0.000]
Observations	212	
R^2 / R^2 adjusted	0.990 / 0.989	

* *p<0.05* ** *p<0.01* *** *p<0.001*

Table S4 Time Series Analysis with Respondents more likely to get vaccinated if recommended by friends and family as Covariate.

	**Vaccination Acceptance Model 1**		**Vaccine Acceptance Model 2**	
Predictors	*Estimates*	*CI*	*Estimates*	*CI*
Trust (lag 1)	0.730 ** (-0.272)	[0.191, 1.269]		
Trust (lag 2)	-0.262 (-0.293)	[-0.840, 0.317]		
Trust (lag 3)	0.039 (-0.293)	[-0.541, 0.618]		
Trust (lag 4)	0.456 (-0.254)	[-0.047, 0.959]		
Net Sentiment (lag 1)			0.728 ** (-0.261)	[0.212, 1.244]
Net Sentiment (lag 2)			-0.433 (-0.287)	[-1.001, 0.136]
Net Sentiment (lag 3)			0.167 (-0.287)	[-0.400, 0.735]
Net Sentiment (lag 4)			0.151 (-0.245)	[-0.334, 0.635]
Vaccination Acceptance with Family Recommendation (lag 1)	-0.002 (-0.057)	[-0.115, 0.112]	0.003 (-0.058)	[-0.112, 0.118]
Vaccination Acceptance with Family Recommendation (lag 2)	-0.028 (-0.062)	[-0.150, 0.094]	-0.021 (-0.062)	[-0.144, 0.102]
Vaccination Acceptance with Family Recommendation (lag 3)	-0.052 (-0.06)	[-0.171, 0.067]	-0.029 (-0.062)	[-0.150, 0.093]
Vaccination Acceptance with Family Recommendation (lag 4)	0.003 (-0.056)	[-0.108, 0.114]	-0.023 (-0.057)	[-0.135, 0.089]
trend	0.001 *** (0)	[0.000, 0.001]	0.000 ** (0)	[0.000, 0.001]
Observations	145		145	
R^2 / R^2 adjusted	0.892 / 0.881		0.889 / 0.878	

* *p<0.05* ** *p<0.01* *** *p<0.001*
